# Structural Characteristics and Properties of the RNA-Binding Protein hnRNPK at Multiple Physical States

**DOI:** 10.3390/ijms26031356

**Published:** 2025-02-05

**Authors:** Quang D. Le, Amanda Lewis, Alice Dix-Matthews, Philippe Ringler, Anthony Duff, Andrew E. Whitten, Rob Atkin, Manuel Brunner, Diwei Ho, K. Swaminathan Iyer, Andrew C. Marshall, Archa H. Fox, Charles S. Bond

**Affiliations:** 1 School of Molecular Sciences, University of Western Australia, Crawley, WA 6009, Australia; lequangdung@hus.edu.vn (Q.D.L.); archa.fox@uwa.edu.au (A.H.F.); 2 Faculty of Biology, VNU University of Science, 334-Nguyen Trai Street, Ha Noi 100000, Vietnam; 3 Center for Cellular Imaging and NanoAnalytics (C-CINA), Biozentrum, University of Basel, 4001 Basel, Switzerlandphilippe.ringler@unibas.ch (P.R.); 4 Australian Centre for Neutron Scattering, Australian Nuclear Science and Technology Organisation, New Illawarra Road, Lucas Heights, NSW 2234, Australia; 5 School of Human Sciences, University of Western Australia, Crawley, WA 6009, Australia

**Keywords:** hnRNPK protein, material state transitions, monomer, aggregate, phase separation, hydrogel, environmental factors, protein characteristics

## Abstract

Heterogeneous nuclear ribonucleoprotein K (hnRNPK) is an RNA-binding protein containing low-complexity domains (LCDs), which are known to regulate protein behavior under stress conditions. This study demonstrates the ability to control hnRNPK’s transitions into four distinct material states—monomer, soluble aggregate, liquid droplet, and fibrillar hydrogel—by modulating environmental factors such as temperature and protein concentration. Importantly, the phase-separated and hydrogel states are newly identified for eGFP-hnRNPK, marking a significant advancement in understanding its material properties. A combination of biophysical techniques, including DLS and SEC-LS, were used to further characterize hnRNPK in monomeric and soluble aggregate states. Structural methods, such as SANS, SAXS, and TEM, revealed the elongated morphology of the hnRNPK monomer. Environmental perturbations, such as decreased temperature or crowding agents, drove hnRNPK into phase-separated or gel-like states, each with distinct biophysical characteristics. These novel states were further analyzed using SEM, X-ray diffraction, and fluorescence microscopy. Collectively, these results demonstrate the complex behaviors of hnRNPK under different conditions and illustrate the properties of the protein in each material state. Transitions of hnRNPK upon condition changes could potentially affect functions of hnRNPK, playing a significant role in regulation of hnRNPK-involved processes in the cell.

## 1. Introduction

The intracellular environment is densely packed with biological materials [1], leading to macromolecular crowding effects that influence protein structure, function, behavior, and stability [2]. Proteins exhibit dynamic physical states that can change under varying conditions [3], allowing functional flexibility [4]. These transitions, which depend heavily on environmental factors [5,6], enable proteins to shift from their native state to various forms, such as solid-like aggregates, gels, or liquid-like droplets [7,8,9]. This adaptability helps protect proteins from degradation [10] and allows them to function in membraneless compartments or organelles [11,12,13]. Despite these transitions, proteins are prone to aggregation under stress. To counter this, cells employ molecular chaperones to assist in protein folding and maintain their native states [14,15]. Additionally, low-complexity domains (LCDs) play a key role in promoting transitions that protect proteins under changing conditions such as temperature, concentration, or pH [16,17].

**Figure 1:**
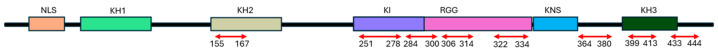
Domain map of full-length hnRNPK used in this study: NLS (amino acids 21–37), KH1 (amino acids 46–98), KH2 (amino acids 149–197), KI (amino acids 236–273), RGG (amino acids 236–335), KNS (amino acids 338–361), KH3 (amino acids 391–439), and sequences of hnRNPK low-complexity domains in red arrows (bottom of Figure 1).

Heterogeneous nuclear ribonucleoprotein K (hnRNPK), a DNA- and RNA-binding protein [18,19], is abundant in the nucleus, cytoplasm, and mitochondria [20]. hnRNPK contains low-complexity domains (LCDs), identified using the SEG algorithm [21] from PlaToLoCo [22]. As a central signaling hub, hnRNPK regulates various cellular processes through its multiple domains, including three K-homologous (KH) domains, an RGG domain, and a K-interactive (KI) region [23] (Figure 1). hnRNPK has been observed in three material states: monomer, soluble aggregate (uncharacterized), and KH3 domain crystals [24,25].

Environmental stressors, such as heat shock and mutations, can cause proteins to adopt abnormal conformations, exposing hydrophobic regions and leading to irreversible aggregation [26]. The aggregation propensity varies significantly among different proteins. RNA-binding proteins with low-complexity domains (LCDs), such as FUS and TDP-43, are particularly prone to aggregation and are linked to neuromuscular disorders like amyotrophic lateral sclerosis (ALS) [27].

LCDs are regions in proteins composed of a limited subset of amino acids, often resulting in intrinsically disordered structures [28]. These domains can initiate protein aggregation in vitro, as observed with the LCD fragments of U1-70K [29]. In addition to aggregation, LCDs can facilitate liquid–liquid phase separation, underscoring their role in the phase behavior of RNA-binding proteins, which is crucial for the formation of membraneless organelles [9,30]. Moreover, LCDs alone can drive the formation of protein hydrogels, as demonstrated by the prion-like LCDs of FUS and RBM14 forming amyloid-like fibrils under specific conditions [17,31].

This study investigates the transitions of hnRNPK, a protein containing both nucleic acid-binding domains and low-complexity domains (LCDs) that are prone to aggregation, phase separation, and gelation [29,30,31,32,33]. Previous research focused on stabilizing hnRNPK to prevent aggregation, but provided limited insights into its properties and transition from monomer to aggregate [24]. Herein, we employed dynamic light scattering (DLS) to measure changes in hnRNPK’s hydrodynamic diameter, revealing that aggregation is a dynamic process influenced by temperature and protein concentration. Light scattering and TEM further characterized the distinct monomeric and aggregated states of hnRNPK, illustrating the measured stoichiometry of novel structures of the hnRNPK monomer and aggregates. Additional small-angle X-ray scattering (SAXS) and small-angle neutron scattering (SANS) experiments provided structural details, showing an elongated monomer shape.

Notably, at low temperatures and high concentrations, eGFP-hnRNPK formed a hydrogel with an amyloid-like fibril structure, confirmed by scanning electron microscopy (SEM) and X-ray diffraction. In the presence of PEG 8000, GFP-hnRNPK phase-separated into liquid droplets, observed via fluorescence microscopy. Further experiments demonstrated the vital role of PEG 8000 and salt concentrations on the phase behavior of hnRNPK.

Collectively, these results demonstrated the complicated behaviors of hnRNPK, resulting in four distinct material states: soluble soluble monomer, soluble aggregate, liquid condensate, and hydrogel. The emergence of two novel states, hydrogel and liquid condensate, together with structural characterization of monomeric and aggregate states of hnRNPK, suggests the exciting potential of hnRNPK transitions as a cellular mechanism to regulate the protein’s functions in the cell.

## 2. Results

### 2.1. The Unstable Nature of hnRNPK

Prior research has reported on the instability of the hnRNPK protein and its tendency to aggregate in solution [24]. The same aggregation phenomenon was observed during hnRNPK purification in this study. To identify what factors induce hnRNPK aggregation, DLS was employed to investigate the solution properties of hnRNPK over time and in varying conditions by monitoring the change in the hydrodynamic diameter of the hnRNPK protein. hnRNPK is unstable and quickly aggregates immediately after secondary purification using SEC; therefore, freshly purified hnRNPK protein was used to minimize the impact of swift aggregation. To avoid variation between hnRNPK purification, all hnRNPK samples used for DLS experiments came from the same batch of purification.

**Figure 2:**
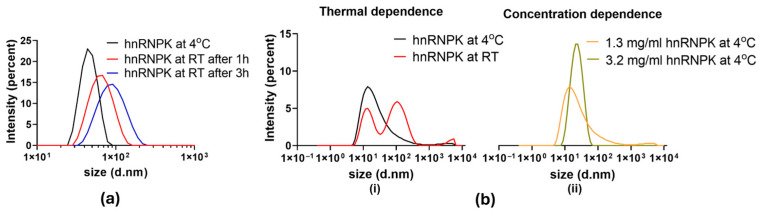
DLS measurement of the hydrodynamic diameter of hnRNPK derived from size distribution by intensity. (**a**) hnRNPK aggregation over time. DLS traces of 0.5 mg/mL hnRNPK at 4 °C, at 25 °C after 1 h, and at 25 °C after 3 h. (**b**) Impact of temperature and concentration on hnRNPK aggregation. (**i**) DLS traces of 1.3 mg/mL hnRNPK at 4 °C and at 25 °C after 2.5 h. (**ii**) DLS traces of 1.3 mg/mL and 3.2 mg/mL hnRNPK at 4 °C.

DLS analysis revealed the behavior of hnRNPK upon temperature change. At 4 °C, a 0.5 mg/mL hnRNPK sample had a peak at 43.8 nm (Figure 2a-black line). The same sample incubated at room temperature for one and three hours showed an increase in particle size to 68.1 nm (Figure 2a-red line) and 91.3 nm (Figure 2a-blue line), respectively, with reduced peak height and increased peak width. These results show a clear increase in the diameter of hnRNPK particles upon raising the temperature.

Having observed the instability of hnRNPK at room temperature, we next used DLS to further quantify the impact of temperature and concentration on freshly purified hnRNPK protein in order to obtain better insight into behaviors of hnRNPK protein upon condition changes.

At 4 °C, a freshly purified hnRNPK sample at 1.3 mg/mL had one single peak at 13.5 nm in diameter, followed by a long tail and a small peak of aggregate at 3580.0 nm. At 4 °C, the freshly purified hnRNPK protein was substantially smaller (13.5 nm). Upon raising the temperature to 25 °C, the peak at 13.5 nm was reduced in height, indicating a reduction in the number of particles at this size. At the same time, a major second peak was observed at 105.7 nm, together with another small peak of aggregate at 5560.0 nm (Figure 2b(i)), with clear resolution between three peaks. Intriguingly, hnRNPK protein aggregates changes from a single peak to bimodal distribution in Figure 2b(i), while the shift is monomodal distribution in Figure 2a, suggesting that hnRNPK protein might assume different modes of self-association during the aggregation process. These DLS data indicate the impact of temperature on hnRNPK, increasing the size of hnRNPK particles over time.

The same protein sample was concentrated from 1.3 mg/mL to 3.2 mg/mL to assess the impact of protein concentration on hnRNPK aggregation. DLS measurements revealed a similar increase in size of hnRNPK upon rising protein concentration at 4 °C. After being concentrated from 1.3 mg/mL to 3.2 mg/mL, the main peak moves from 13.5 nm to 21.0 nm with higher peak height and becomes symmetrical as the long tail from the 1.3 mg/mL hnRNPK sample disappears (Figure 2b(ii)). Both samples have small aggregate peaks at 3580 nm for the 1.3 mg/mL sample and 5560 nm for the 3.2 mg/mL sample. The substantial increase in peak height at higher protein concentration indicates the existence of larger species, resulting in stronger scattering intensity. The DLS analysis unraveled the significant impact of temperature and concentration as two key factors on hnRNPK’s behavior, driving the protein into aggregation upon condition changes.

### 2.2. Monomeric State of hnRNPK

Based on our finding that the temperature and concentration can drive hnRNPK into aggregation, we therefore designed a series of experiments using low concentrations of hnRNPK at low temperature to characterize hnRNPK protein in its monomeric state.

**Figure 3:**
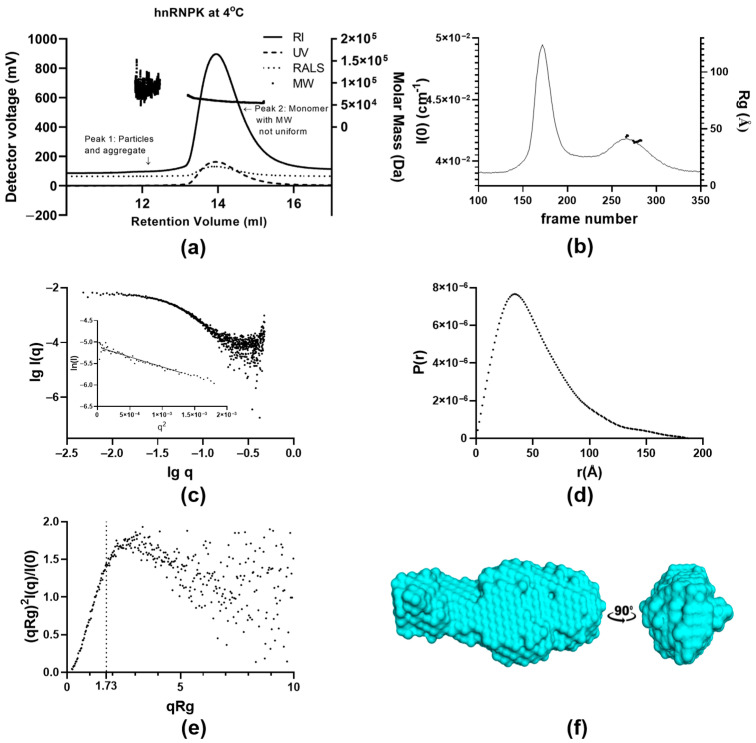
Stoichiometry and structure of hnRNPK at 4 °C. (**a**) SEC-LS of hnRNPK at 4 °C with RI, UV, RALS traces, and molecular mass distribution. (**b**) Plot of *I*(0) trace for the SEC-SAXS of hnRNPK at 4 °C. (**c**) Log I(q) vs. log q plot and Guinier plot. (**d**) The *P(r)* profile. (**e**) Dimensionless Kratky plot of the scattering data. (**f**) Ab initio model of hnRNPK at 4 °C.

We first used SEC-LS at room temperature to determine the molar mass (MM) and homogeneity of hnRNPK particles when the hnRNPK sample was kept at 4 °C. The theoretical MM of the full-length wild-type hnRNPK monomer with an additional glycine residue at the N-terminus, used in this study, was 51,033 Da. SEC-LS analysis can be used to compare the measured MM of hnRNPK to the theoretical MM of the measured samples and reveal the stoichiometry of the hnRNPK sample kept at 4 °C. The elution profile displays a small shoulder, followed by a major peak with good resolution (Figure 3a). The small shoulder (peak 1) is eluted at 12.4 mL with an estimated MM of 101.32 kDa (Table 1). The MM distribution shows a wide range (49.0–152.9 kDa), most probably representing a small number of protein stoichiometries (one to three molecules). The major peak (peak 2) elutes at 13.9 mL, exhibiting a MM value of 59.1 kDa (Table 1), suggesting the majority of particles in peak 2 are hnRNPK monomers. The molar mass points, distributed linearly across the major peak, range from 54.0 kDa to 73.0 kDa. This MM distribution indicates homogeneity of hnRNPK as the protein was kept at low temperature and low concentration. A slight slope of MM distribution across peak 2 indicates that in this condition, there is still a small amount of hnRNPK aggregate coexisting together with the majority of hnRNPK monomer in solution. In combination with DLS, SEC-LS showed that 4 °C and low protein concentration was sufficient to maintain the majority of hnRNPK in the monomeric state. Based on these results, we designed SAXS and negative-stain transmission electron microscopy (TEM) experiments in optimal conditions to study the structure of hnRNPK monomer.

Using SEC-SAXS (Table 2), we observed that while the protein sample was kept at 4 °C, it displayed heterogeneity, as both aggregate and monomer were present in the sample. The chromatography trace following *I*(0) for the separation of 6.2 mg/mL hnRNPK at 4 °C shows two well-separated peaks (Figure 3b). The first peak, with high intensity, is due to large polydispersed aggregated protein, as no *Rg* could be established from the scattering data. The second peak, on the other hand, contains scattering data that can be used for further analysis when background-subtracted using buffer-only frames from an appropriate part of the experiment. The MM distribution across peak 2 varies from 40 to 210 kDa, suggesting the presence of multiple oligomers along with monomeric hnRNPK (Figure S1—Supplementary data). The MM variation in hnRNPK at 4 °C from SAXS resembles such values observed in SEC-LS, ranging from 49.0 to 152.9 kDa (Table 2). SAXS samples were transported to the Australian Synchrotron. The higher MM of hnRNPK measured from SAXS is perhaps due to the effect of freeze–thaw cycles in comparison to freshly purified hnRNPK immediately used after purification for SEC-LS experiments at 4 °C. The MM distribution of hnRNPK at 4 °C measured by SAXS and SEC-LS indicates hetero-oligomerisation of hnRNPK, while multiple oligomeric states of hnRNPK coexist with monomeric hnRNPK in solution.

SEC-SAXS can resolve heterogeneous samples into multiple populations of elution. The scattering data, from frames 360–379 (buffer), and 273–283 (protein sample), were averaged to study hnRNPK monomers at 4 °C. By studying this trait of SEC-SAXS, the structure of the homogeneous population of interest in solution can be determined [34]. Due to complex behaviors of hnRNPK samples at 4 °C, selecting appropriate scattering frames is a critical step in analyzing the solution structure of monomeric hnRNPK, eliminating any sudden increase in MM value to ensure the homogeneity of hnRNPK monomers [35].

The log I(q) vs. log q plot shows the X-ray scattering of hnRNPK particles in the averaged frames from the second peak. The plot is linear and approaching the y-axis horizontally at low angles (Figure 3c). In addition, the Guinier plot of the scattering data displays linear behavior (inset-Figure 3c), despite a minimal uptick at the lowest resolution, most probably due to the presence of some large particles.

The MM, estimated by Bayesian probability calculation, is one of the primary parameters derived from SAXS analysis to demonstrate the solution state of protein samples [35]. The estimated MM of the hnRNPK sample was 54.4 kDa (Table 2) with 6.7% deviation from the theoretical MM of the hnRNPK monomer, confirming this set of scattering data derived from hnRNPK particles in their monomeric state.

The *P(r)* distribution shows a smooth, concave curve with a peak at approximately 4.0 nm and an extended tail that approaches zero at *D*_max_ of 18.7 nm (Figure 3d). This distribution indicates that the hnRNPK monomer in solution adopts a globular, elongated shape. The interpretation of the extended tail depends on whether higher oligomeric species are contaminating the hnRNPK monomer. In the absence of contamination, given that the selected scattering frames have estimated molecular masses closely aligned with the theoretical molecular mass of the hnRNPK monomer (51 kDa), the globular, compact particles would exhibit a bell-shaped *P(r)* distribution, while the extended tail could be attributed to the flexibility of unfolded particles. Despite careful selection of scattering frames to focus on hnRNPK monomers, the low resolution of size-exclusion chromatography implies potential contamination by higher oligomeric hnRNPK species, with estimated molecular masses ranging from 40 to 210 kDa (Figure S1). The presence of a few hnRNPK oligomers could also explain the extended tail from 11 to 18 nm in the *P(r)* distribution.

The dimensionless Kratky plot initially rises sharply to a peak at low *qR_g_* (ca. 1.8), and then gradually drops down toward the x-axis, suggesting that the hnRNPK monomer possesses an ordered structure with a degree of flexibility (Figure 3e). The plot becomes too noisy to interpret above the *qR_g_* value of 5.

From the scattering data, 20 independent bead models were generated using DAMMIF program [36]. The final normalized spatial discrepancy (NSD) was 0.651 (Table 2), which suggests that most of the models are very similar in shape. The 20 models were averaged using DAMAVER [37], producing a representative ab initio model of hnRNPK monomer with elongated shape as shown in Figure 3f. Moreover, the χ^2^ of 0.26 (Table 2), indicates an extremely good fit between the model and scattering data (the error model employed at the SAXSWAXS beamline of the Australian Synchrotron resulted in a χ^2^ of 0.25, representing ideal fit). The ab initio model in Figure 3f is the most accurate solution structure representing either flexible hnRNPK monomers or hnRNPK monomers with mixed scattering signals from a few higher-MM hnRNPK oligomers.

**Figure 4:**
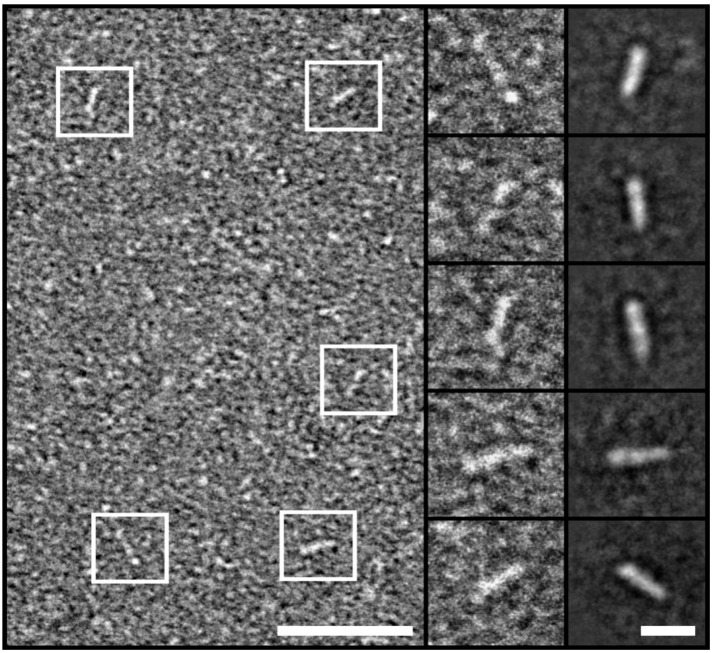
Structural analyses of hnRNPK by negative-stain transmission electron microscopy. Left: Overview image showing well-distributed particles. Five particles are highlighted (white boxes), scale bar: 50 nm. Insets: First column: 2× enlarged views of the 5 representative particles highlighted in the corresponding overview. Second column: 5 2D class averages, each obtained by averaging about 20 particles. Scale bar: 10 nm, all inset boxes are 25 by 25 nm.

To further characterize the morphology of the hnRNPK monomer, we carried out TEM experiments beginning with a low concentration of hnRNPK protein. The preparation of TEM grids with hnRNPK protein was performed on ice to minimize hnRNPK aggregation. TEM images show that at the low concentration of 2.48 µM (ca. 0.13 mg/mL), hnRNPK protein particles were well distributed over the grids. The overview panel of Figure 4 reveals raw hnRNPK particles, which are highlighted in white boxes. The contrast of the images is sufficient to identify and select raw hnRNPK particles from the background, as shown in the first column of Figure 4. From the raw particles, 2D averaging was performed to produce five distinct 2D class averages with elongated shapes, with each averaged from around 20 raw particles. The length of these five 2D averages ranges from 10.8 nm to 13.5 nm. The images suggest a structure with two to three regions of higher electron density, which may reflect the domain organization of hnRNPK. The overall size and shape of the hnRNPK particles imaged by TEM is in agreement with the solution structure of the hnRNPK monomer SAXS profile.

### 2.3. Aggregation State of hnRNPK

Having characterized the properties and structure of the hnRNPK monomer, we next set out to characterize hnRNPK aggregates induced by incubation at room temperature. SEC-LS was again used to investigate the stoichiometry and homogeneity of hnRNPK, while TEM and SAXS experiments were carried out to study the structure of hnRNPK.

**Figure 5:**
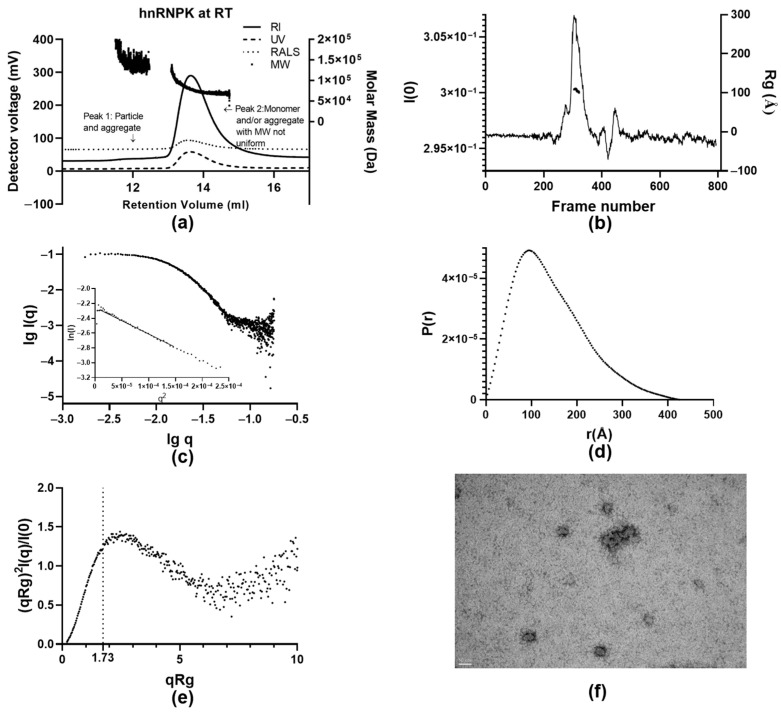
Stoichiometry and solution structure of hnRNPK at room temperature. (**a**) SEC-LS of hnRNPK at room temperature with RI, UV, RALS traces, and molecular mass distribution. (**b**) Plot of *I*(0) trace for the SEC-SAXS of hnRNPK at room temperature. (**c**) Log I(q) vs. log q plot and Guinier plot. (**d**) The *P(r)* profile. (**e**) Dimensionless Kratky plot of the scattering data. (**f**) TEM of hnRNPK aggregates at room temperature, scale bar: 50 nm.

The SEC-LS elution profile of hnRNPK at room temperature indicates an increase of protein aggregates compared to the hnRNPK sample at 4 °C (Figure 3a), as larger species can be observed from the chromatogram. The MM distributions of peak 1 and peak 2 overlap each other with poor resolution between two peaks (Figure 5a). Peak 1 (12.5 mL) had an MM of 146.9 kDa, with MM points varying from 114.8 kDa to 282.4 kDa, strongly suggesting polydisperse aggregation. Similarly, the majority of protein particles in peak 2 (13.6 mL) increase in size in comparison to the sample at 4 °C (13.9 mL) from 59.1 kDa to 78.0 kDa (Table 1). The MM distribution of peak 2 is markedly uneven, as the MM values vary from 138.0 kDa to 51.0 kDa, indicating heterogeneity of the sample across peak 2 with higher oligomeric states of hnRNPK formed due to the effect of room temperature on the samples. Together with DLS, SEC-LS analysis illustrated the impact of temperature on hnRNPK aggregation, establishing the specific condition for us to further study the aggregation process of hnRNPK protein.

Having observed the aggregation of hnRNPK induced by room temperature, we next used SEC- SAXS to investigate the solution structure of hnRNPK aggregates. The frames used to generate data were 51–102 for buffer and 305–321 for protein sample. The *I*(0) trace of hnRNPK at room temperature displays one large peak along three small peaks and a noisy background (Figure 5b), suggesting heterogeneity of hnRNPK at room temperature. The noisy background is perhaps due to a minimal degree of air in the SAXS system.

The log I(q) vs. log q plot, the primary data for hnRNPK at room temperature (Figure 5c), is in linear form and horizontal at low log q values. The scattering data show linear behavior in the Guinier region (inset, Figure 5c).

The *P(r)* distribution shows a maximum peak at 10.0–11.0 nm and approaches the x-axis smoothly at *D*_max_ at 41.0 nm (Figure 5d), indicating a substantial increase in size in comparison to the protein sample at 4 °C. Despite the fact that the hnRNPK concentration of the room temperature sample was 0.82 mg/mL compared to 6.2 mg/mL of the 4 °C sample, the maximum dimension of the *P(r)* from the room temperature sample (Figure 5d) is much higher than the value of the 4 °C sample (Figure 3d). The increase in dimension and area under the *P(r)* curve, even at lower protein concentration, is clear evidence of hnRNPK aggregation at room temperature.

The Kratky plot shows a maximum value of 1.4 at a *qR_g_* value of 2.5, dropping down toward the x-axis to form a partly bell shape before rising at a *qR_g_* value of 7 (Figure 5e). Given the large size of hnRNPK aggregates, the Kratky plot most probably represents a globular oligomeric structure with some degree of polydispersity.

The established molecular mass of hnRNPK particles at room temperature was 873.1 kDa (Table 2), evidently showing aggregate of hnRNPK formed in the solution. An attempt was made to generate an ab initio model of the hnRNPK aggregate; however, the final NSD was 1.1, suggesting a wide variety of classes from the calculations (Table 2). This result indicates the high polydispersity of hnRNPK aggregates, which makes the bead modeling an inappropriate approach to illustrate the structure of hnRNPK aggregates in solution.

In order to further characterize the morphology of the hnRNPK aggregates, TEM was performed using TEM grids of hnRNPK prepared at room temperature to visualize the effect of temperature on the overall shape of hnRNPK protein. The TEM images display hnRNPK aggregates as several separated clumps and a big cluster with diameters varying from 22 nm to 200 nm (Figure 5f), which is in agreement with the SAXS analysis of hnRNPK aggregates showing a highly polydisperse protein sample in solution. This is consistent with the highly uneven molar mass distribution of hnRNPK recorded by the SEC-LS result, showing the coexistence of multiple higher-order hnRNPK species in solution at room temperature.

### 2.4. Characterization of hnRNPK Monomers in Mixture with Aggregates

Having established the structures of hnRNPK monomer and aggregate, next we performed a SANS experiment to further characterize the structure of monomeric hnRNPK within heterogeneous samples. SANS relies on contrast variation, mixing hydrogenated and deuterated hnRNPK (H-hnRNPK:D-hnRNPK) at a certain ratio (H:D), enabling us to determine the structure of hydrogenated components within a multi-component complex. Given the negative impact of high temperature and concentration on hnRNPK monomers, hydrogenated hnRNPK was purified at 4 °C and maintained at a concentration of 1.1 mg/mL to minimize aggregation. The optimal H:D ratio was 1:25 with 1.1 mg/mL of hydrogenated hnRNPK mixed with deuterated hnRNPK to achieve a total concentration of 26.6 mg/mL. While the low concentration of 1.1 mg/mL minimized aggregation of H-hnRNPK, it was sufficiently strong to extract the scattering signal of the hydrogenated monomer from the mixture. At the same time, our previous DLS analysis at room temperature suggested that at 20 °C, it is highly possible the 26.6 mg/mL H-hnRNPK:D-hnRNPK mixture will form aggregates due to the impact of high concentration and temperature.

**Figure 6:**
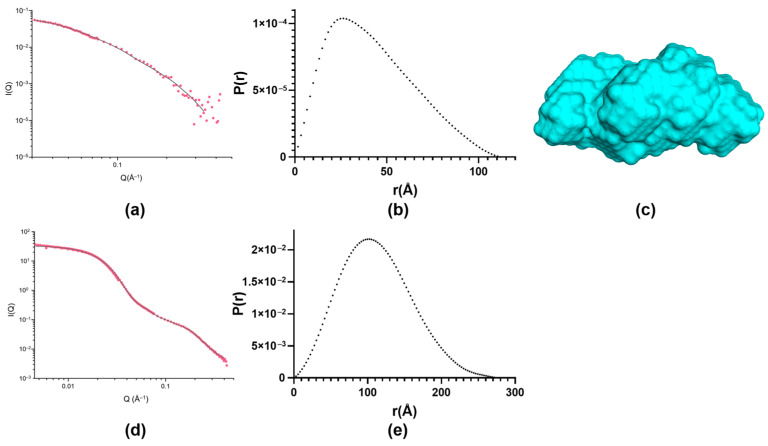
Solution structure of hnRNPK derived from SANS. (**a**) Background-subtracted SANS data for hydrogenous and deuterated hnRNPK (H:D = 1:25) in deuterated buffer with 1.1 mg/mL of unmatched hnRNPK in a total concentration of 26.6 mg/mL (**b**) The *P(r)* profile of 1:25 (H:D) hnRNPK sample. (**c**) Ab initio model of hnRNPK monomer from SANS profile. (**d**) Background-subtracted SANS data for hydrogenous hnRNPK in deuterated buffer at a concentration of 28.9 mg/mL (**e**) *P(r)* profile of hnRNPK aggregates.

SANS analysis (Figure 6a) shows the background-subtracted SANS data for hnRNPK from the 1:25 (H:D) mass ratio sample. To prevent scattering signals from protein aggregates overlapping the signals from the hydrogenous hnRNPK sample, the bulk of the protein present was deuterated and contrast-matched in buffers containing ~100% D_2_O. Hydrogenous protein was visible in D_2_O solutions at a concentration of 1.1 mg/mL, and thus the resulting scattering was from the individual hydrogenous hnRNPK particles distributed randomly throughout the deuterated hnRNPK bulk condensate. In addition, using unmatched buffers, we were able to measure the scattering of the whole aggregate.

The scattering profile of the 1:25 (H:D) sample in the low *q* region under 0.02 Å^−1^ was relatively linear. The estimated molecular mass (MM) of protein measured from SANS data was approximately 67.5 kDa (Table 2) [38,39]. The measured MM of the hydrogenous hnRNPK particles was close to the theoretical MM of the hnRNPK monomer at 51,033 Da, confirming that the H-hnRNPK maintained at low temperature and low concentration was indeed an hnRNPK monomer. The *P(r)* distribution of H-hnRNPK has a concave curve shape, with a peak at 25–30 Å and a long smooth tail approaching the x-axis (Figure 6b). The *P(r)* profile of the H-hnRNPK suggests an elongated shape for H-hnRNPK protein, which is slightly more compact compared to the hnRNPK monomer measured in the SAXS experiment, with *D*_max_ from SANS and SAXS 11.2 nm and 18.7 nm, respectively (Table 2).

The program DAMMIF [36] was used to generate 20 independent ab initio models from the scattering data, revealing a uniform elongated shape of hnRNPK monomers for 20 models. The final normalized spatial discrepancy (NSD) was 0.893, which is relatively high (Table 2), suggest that the reconstruction was moderately stable and likely represented reliable shape determination. Models were averaged using DAMAVER [37].

SANS analysis of hnRNPK aggregates was carried using 28.9 mg/mL hydrogenated hnRNPK protein. Figure 6d shows the background-subtracted SANS data for hnRNPK in deuterated buffer solution. The scattering data have been reduced to log[*I*(*q*)] vs. log(*q*), descending gradually in the low *q* region. Such a scattering profile suggests aggregate formation in the hnRNPK sample [40]. In addition, the estimated molecular mass (MM) of the 28.9 mg/mL hnRNPK sample measured from SANS data was approximately 1267.9 kDa (Table 2) [38,39]. The high measured MM of the sample confirmed aggregation of the sample at high concentration.

The *P(r)* profile of hnRNPK aggregates had a relatively symmetrical shape, with a short tail approaching the x-axis at 28–29 nm (Figure 6e). The *P(r)* distribution of hnRNPK aggregates is in agreement with SASView analysis, suggesting an overall ellipsoidal shape of hnRNPK aggregates with a *D*_max_ value of 28.6 nm (Table 2). Given the high concentration of the hnRNPK sample (28.9 mg/mL) and the ambient temperature conditions, the hnRNPK aggregates are likely to exhibit significant heterogeneity. Consequently, further solution structure analysis of the hnRNPK aggregates using DAMMIF was not pursued in the SANS experiment.

The SANS analysis was in agreement with the DLS data, showing the negative impact of high temperature and concentration on hnRNPK aggregation over time. Collectively, our DLS and SANS data illustrate the molecular basis of hnRNPK transition from monomer to aggregate. Specifically, SANS data suggested that H-hnRNPK monomers retained their elongated structure when forming larger aggregates due to the impact of the 20 °C temperature and the high concentration of 26.6 mg/mL of the 1:25 (H:D) mixture. Although the SANS result suggested a similarly elongated shape of hnRNPK monomers, the *D*_max_ value recorded by SANS was only 11.2 nm, substantially shorter than the values recorded by TEM (up to 13.5 nm) and SAXS (18.7 nm) (Table 2). This shorter *D_max_* of SANS may be the result of a large compaction from aggregates on hnRNPK monomers, reducing the length of the monomers. SANS data for hnRNPK aggregates show an averaged structure with *D*_max_ value of 28.6 nm. Consistently with the SAXS data and TEM results for hnRNPK aggregates, the SANS model again demonstrates that hnRNPK aggregation is an ever-changing process, resulting in various sizes of hnRNPK aggregates.

### 2.5. Hydrogel of hnRNPK

**Figure 7:**
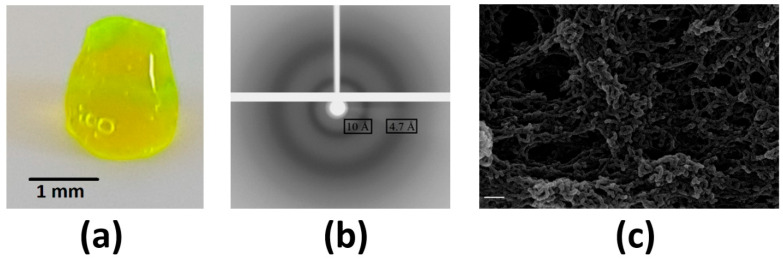
Gelation of eGFP-hnRNPK induced at low temperature and high protein concentration. (**a**) eGFP-hnRNPK in hydrogel form. Bar 1 mm. (**b**) X-ray diffraction of eGFP-hnRNPK hydrogel with typical amyloid rings at 4.7 Å and 10 Å. (**c**) SEM image of eGFP-hnRNPK hydrogel showing fibrillar structure. Bar 200 nm.

hnRNPK contains several low-complexity domains (LCDs) within its sequence, as demonstrated in Figure 1. Given the ability of LCDs in driving hydrogel formation in protein, such as FUS and hnRNPKA2 in vitro [41,42], hnRNPK was tested to ascertain whether it could form hydrogel, as with other proteins possessing prion-like LCDs. After being purified, concentrated, and dialyzed in gel filtration buffer overnight at 4 °C, gelation of eGFP-hnRNPK occurred, as shown in Figure 7a. Besides two common material states, monomer and aggregate, hydrogel is a novel material state of hnRNPK observed in in vitro assays. Further experimentation to study the biophysical properties of eGFP-hnRNPK hydrogel was performed using X-ray diffraction, which revealed two diffraction rings at 4.7 Å and 10 Å (Figure 7b). These two diffraction patterns are characteristic signals of the cross-β structure existing in amyloid fibrils [43]. In addition, SEM also displays a fibrillar mesh surface of eGFP-hnRNPK (Figure 7c). Together, these data reveal for the first time the ability of eGFP-hnRNPK to form a fibrillar hydrogel in vitro with amyloid-like structure.

### 2.6. Phase Separation of hnRNPK

**Figure 8:**
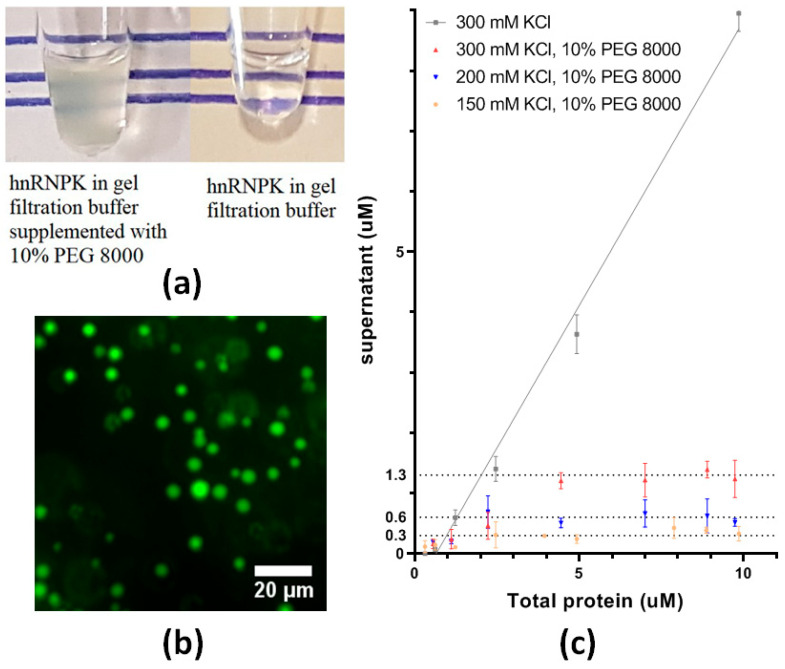
Liquid–liquid phase separation of eGFP-hnRNPK. (**a**) Addition of 10% PEG 8000 induced phase separation of hnRNPK. (**b**) Fluorescence microscopy of eGFP-hnRNPK droplets induced by 10% PEG 8000 revealed liquid droplets. (**c**) Measurement of the saturation concentration of eGFP-hnRNPK phase separation induced at three different salt concentrations.

LCDs have been linked to liquid–liquid phase separation (LLPS) of proteins, where the protein can partition into a condensed phase, forming liquid-like droplets. LLPS is commonly observed in nuclear RNA-binding proteins, and is implicated in the formation of membraneless organelle structures in the cell nucleus, including nucleoli and paraspeckles, of which hnRNPK is a component. To explore the potential of hnRNPK to phase-separate, we used a crowding agent, PEG 8000, to induce phase separation of eGFP-hnRNPK. In experiments carried out on ice, dilution of eGFP-hnRNPK into gel filtration buffer at various KCl concentrations produced clear solutions. In contrast, the addition of 10% PEG 8000 in gel filtration buffer turned eGFP-hnRNPK solution turbid, indicating the protein had precipitated in some form (Figure 8a). Fluorescence microscopy of eGFP-hnRNPK confirmed the phenomenon as LLPS, with the formation of spherical droplets suspended in the solution (Figure 8b).

Salt concentration has a crucial role in protein phase separation, influencing phase behaviors of proteins as the salt concentration varies in the solution. To further quantify the effect of PEG 8000 and KCl concentration on eGFP-hnRNPK phase separation, we carried out a centrifuge assay to assess the phase separation induced by PEG 8000 at different KCl concentrations. Upon phase separation, eGFP-hnRNPK coexisted in two separated phases, a dilute phase and a dense phase, in the solution. Centrifugation sedimented the dense phase from the protein-depleted dilute phase. We measured the concentration of eGFP-hnRNPK in the dilute phase, also known as the saturation concentration (*C*_sat_), to evaluate the phase separation of eGFP-hnRNPK in different conditions. In the presence of PEG 8000, eGFP-hnRNPK in all three conditions phase-separated to different degrees (Figure 8c). Specifically, eGFP-hnRNPK diluted in gel filtration buffer alone (■) showed no sign of phase separation with the measured protein concentration of ca. 10 µM (Figure 8c). In the presence of 10% PEG 8000 (▲), a substantial reduction in hnRNPK concentration in the supernatant to 1.3 µM resulted in a high level of phase separation induced by PEG 8000. Strikingly, the decrease in KCl concentration to 200 mM (▼) and 150 mM (⬤) reduced the *C*_sat_ of eGFP-hnRNPK in the two conditions to 0.6 µM and 0.3 µM, respectively (Figure 8c). The variety of *C*_sat_ at different KCl concentrations indicated the impact of the salt concentration in the phase separation of eGFP-hnRNPK. The lowest KCl concentration at 150 mM depleted the most eGFP-hnRNPK protein from the dilute phase into the dense phase. In comparison, higher KCl concentrations in the other two conditions, 200 mM and 300 mM KCl, allowed more eGFP-hnRNPK protein to remain in the dilute phase. Various phase behaviors of eGFP-hnRNPK at different salt concentrations proved the significance of a stable physiological salt concentration in the cells. Stress may change this balance in the cell, which in turn depletes eGFP-hnRNPK in cellular processes.

## 3. Discussion

Proteins have been found to exist in multiple material states. The transitions of protein monomers to other physical states, such as protein aggregates or liquid–liquid droplets, play significant roles in the pathogenesis of several neurodegenerative conditions [44,45]. Prior research has demonstrated hnRNPK aggregation in vitro and successfully used stabilizing additives to reverse and protect hnRNPK monomers from aggregation, with the stoichiometry of hnRNPK monomers confirmed by AUC [24]. In this study, we have demonstrated the substantial impact of temperature and concentration on hnRNPK aggregation. By controlling these two factors, we were able to achieve an optimized condition maintaining freshly purified hnRNPK monomers at 4 °C and low concentration to partially prevent hnRNPK aggregation. The beneficial effect of 4 °C and low protein concentration enabled us to go beyond stoichiometry values, determining the structure of hnRNPK monomers from highly heterogeneous hnRNPK samples. The measured stoichiometry data for hnRNPK by SEC-LS at 4 °C confirmed the monomeric state of hnRNPK, while TEM and SAXS revealed the novel structure of full-length hnRNPK monomers as elongated shape particles.

The structure of hnRNPK monomers was investigated using three distinct techniques in our study, TEM, SAXS, and SANS. Consistently, data from all three approaches reported a similar morphology of hnRNPK proteins as elongated-shape particles. Despite the consistent morphology, the maximum dimensions of hnRNPK measured by TEM, SAXS, and SANS varied considerably: 11.2 nm (SANS), 18.7 nm (SAXS), and 10.8–13.5 nm (TEM). The variation in *D*_max_ might have been due to different hnRNPK conditions causing different extents of interparticle interaction between hnRNPK proteins. In addition, during TEM grid preparation, dehydration of samples can lead to protein shrinkage and subsequently reduce protein length in comparison to *D*_max_ values measured by SAXS [46]. The beneficial impact of low temperature and concentration sustained part of the highly heterogeneous hnRNPK sample in monomeric form, indicated by the small peak following a strong aggregation peak in the SAXS profile of hnRNPK samples at 4 °C (Figure 3b). The heterogeneity of hnRNPK samples at 4 °C, as shown in the aggregation peak in SAXS, together with the aforementioned flexibility of hnRNPK possibly caused by LCDs, represents a major hurdle for crystallization of full-length hnRNPK protein. Instead, crystallization of hnRNPK truncates containing individual domains, namely, KH1, KH2, NLS, and KNS, might be a more reliable alternative to avoid the issue of full-length hnRNPK instability. Another approach to solve full-length hnRNPK structure is cryo-EM, now that we have successfully obtained the low-resolution structure of hnRNPK protein using TEM. The addition of the cryo-EM technique might allow us to take a step further toward visualizing the atomic structure of full-length hnRNPK protein.

Despite the beneficial impact of low temperature (4 °C) and low protein concentration, hnRNPK aggregates were detected simultaneously with hnRNPK monomers (Figure 3b). Room temperature and high protein concentration inducing hnRNPK aggregation allowed us to study the volatile changes in hnRNPK protein during aggregation. High temperature and concentration had a significant impact on hnRNPK, increasing both the hydrodynamic size and molecular mass of hnRNPK over time. Additionally, a combination of SAXS and TEM showed large structures of hnRNPK aggregates of various sizes, ranging from 22 to 200 nm. hnRNPK is well recognized for its RNA-binding function, which plays a pivotal role in the formation of membraneless organelles, such as paraspeckles. The interaction between hnRNPK and NEAT1 RNA serves as a critical foundation for paraspeckle assembly [31]. Beyond paraspeckles, hnRNPK also interacts with and is integral to the stress granule localization of SIRLOIN RNA [47], a process similar to the sequestration of biological materials in stress granules by RNA-binding proteins under cellular stress. Notably, liquid–liquid phase separation (LLPS) has been identified as a key mechanism underlying the formation of these membraneless organelles, including paraspeckles and stress granules [48,49]. This study demonstrated the phase separation capability of hnRNPK. The ability of hnRNPK to form highly complex structures, such as aggregates or phase-separated states, may facilitate its high local concentration within these organelles while enabling their reversible dissolution once cellular stress is alleviated.

Phase separation of protein is a crucial process to form membraneless compartments or organelles in the cell, including stress granules and paraspeckles [50,51]. Many proteins containing low-complexity domains (LCDs) have been found in these membraneless organelles [16,29,41]. These proteins can undergo phase separation in vitro upon changes in conditions such as temperature, salt concentration, or crowding agents [33,52,53], for example, FUS, tau, or α-synuclein [54,55]. hnRNPK is an RNA-binding protein with several LCDs in its sequence. This protein has proven to be one of six proteins essential for paraspeckle formation. Indeed, the introduction of PEG 8000 supplemented with protein in solution has resulted in phase separation of eGFP-hnRNPK, a novel state of this protein until now. PEG 8000 has proven its effect on affecting the phase behavior of FUS, facilitating condensation of the full-length FUS protein [56]. The crowding agent PEG 8000 is able to induce phase separation of eGFP-hnRNPK PK, reorganizing the protein into a dilute phase and dense phase with substantial protein concentration difference. Additionally, fluorescence microscopy revealed spherical droplets suspended in solution. The spherical shape caused by surface tension indicates the liquid-like property of the droplets [9]. Salt concentration has a significant impact on phase behaviors of many proteins, inducing phase separation on FUS [57,58], TDP-43 [59,60], and BRD4 proteins [61,62]. Despite the fact that low-salt conditions alone do not lead to eGFP-hnRNPK phase separation, the protein phase separates in a salt concentration-dependent manner in the presence of PEG 8000, reaffirming the importance of salt to hnRNPK phase separation.

The LCDs of RNA-binding proteins have been linked to protein gelation, driving proteins into a hydrogel-like state. Two essential paraspeckle proteins, FUS and RBM14, are among these proteins, forming hydrogel at high concentration and low temperature [31,63]. hnRNPK is another essential paraspeckle protein, containing both LCDs and RNA-binding domains. In our in vitro experiments, eGFP-hnRNPK was observed to adopt a hydrogel form. Additional studies using X-ray diffraction and SEM revealed the amyloid characteristics of eGFP-hnRNPK hydrogel, which are similar to the hydrogels of FUS and RBM14.

Despite the beneficial effect of low temperature and low concentration, hnRNPK aggregation still occurs with high heterogeneity in solution. The heterogeneity of hnRNPK represents a major hurdle for functional and structural studies of the protein. Therefore, additional effort is required to establish an optimal condition favoring hnRNPK monomers over their aggregated form. Here, we also described two novel material states of eGFP-hnRNPK, liquid–liquid phase separation and hydrogel, as well as the properties of hnRNPK protein in each state. Phase separation has a critical role in mediating the formation of membraneless organelles such as paraspeckles [50], while hydrogel formation of proteins is an essential cellular process responsible for both normal physiology and pathology [64]. The ability of hnRNPK to transform from monomers to aggregates, liquid-like droplets, and hydrogel demonstrates the flexibility of hnRNPK to adapt to condition changes, enabling the protein to maintain its functions in a wide range of conditions. Here, we propose a hypothesis that the transitions of hnRNPK protein between different states are protective cellular mechanisms. These mechanisms are employed to regulate multiple hnRNPK-involved cellular processes during stress by transforming hnRNPK protein to appropriate physical states to maintain hnRNPK functions. Therefore, further functional studies of hnRNPK in different physical states—aggregation, phase-separated, and hydrogel—are required to test this theory.

## 4. Materials and Methods

### 4.1. Protein Expression and Purification

Full-length wild-type hnRNPK protein with an additional glycine residue at the N-terminus was tagged at the N-terminus with a 6x-histidine tag (6xHis), followed by eGFP. The resulting 6xHis-eGFP-hnRNPK fusion construct was cloned into the pETM-11 plasmid. The 6xHis-eGFP-hnRNPK protein in hydrogenated media (H-protein) was overexpressed in *Escherichia coli* Rosetta2 (DE3) cells in 500 mL of LB media supplemented with 50 µg/mL kanamycin, 34 µg/mL chloramphenicol, and 0.5 mM IPTG at 25 °C for 16 h.

Expression of the deuterated 6xHis-eGFP-hnRNPK protein (D-protein) was carried out in *E. coli* BL21 (DE3) using a 2 L bioreactor (Real Time Engineering). For D-protein expression, expression media with 90% D_2_O and hydrogenated glycerol (40 g/L) as a carbon source were used to reach a deuteration level of 75%. The expression culture (LB media), supplemented with 50 µg/mL kanamycin, and 34 µg/mL chloramphenicol, was inoculated with 54 mL of starter culture and grown at 37 °C until the optical density at 600 nm reached 37. The expression culture was then induced by 0.5 mM IPTG and incubated at 20 °C for 16 h.

For protein purification, harvested cells were resuspended in lysis buffer (50 mM HEPES, 2 M KCl, 20 mM imidazole, 100 mM dithiothreitol (DTT), EDTA-free protease inhibitor cocktail (Merck, Burlington, MA, USA), pH 7.4) on ice. Suspended cells were lysed using a high-pressure homogenizer (Emulsiflex C5; Avestin). The lysate was clarified by centrifugation at 24,000× *g* at 4 °C for 45 min. The supernatant was filtered through a 0.22 µm syringe filter (Merck Millipore) before being loaded into a 5 mL Hi-trap column charged with NiCl_2_ (GE Healthcare). GFP-hnRNPK was eluted with hnRNPK elution buffer (50 mM HEPES, 2 M KCl, 500 mM imidazole, pH 7.4). Peak fractions were pooled, incubated with His-tagged TEV protease produced in-house, and dialyzed in hnRNPK gel filtration buffer (50 mM HEPES, 300 mM KCl, pH 7.4) supplemented with 1 mM DTT for 16 h at 4 °C. The digest was then passed through a 5 mL Hi-trap column to separate the cleaved hnRNPK from the mixture. HnRNPK was concentrated to 5 mL (Amicon Ultra Centrifugal filter (EMD Millipore)) and applied to a HiLoad 16/60 Superdex 200 preparative-grade column (Cytiva, Marlborough, MA, USA) equilibrated with hnRNPK gel filtration buffer. Purified hnRNPK was concentrated to the desired concentrations for further experiments using a 30 kDa Amicon centrifugal concentrator (Merck Millipore). Purified protein was evaluated by SDS-PAGE stained with Coomassie blue. To achieve 98% deuteration, purified D-protein was dialyzed for 6 h into deuterated size-exclusion buffer using a 10,000 MWCO Slide-A-Lyzer dialysis cassette (Thermo Scientific, Waltham, MA, USA).

### 4.2. Gelation

GFP-tagged hnRNPK was concentrated to 11.5 mg/mL and dialyzed in buffer (50 mM HEPES, 300 mM KCl, pH 7.4) at 4 °C overnight. Dialyzed protein was centrifuged at 3000× *g* at 4 °C for 10 min to separate the soluble fraction and hydrogel. Hydrogel was kept at 4 °C for 48 h in a sealed tube to gelate into a more rigid form.

### 4.3. X-Ray Diffraction

GFP-hnRNPK hydrogel was placed onto a nylon CryoLoop and mounted on an XtaLab Synergy-S X-ray generator (Rigaku Oxford Diffraction). X-ray diffraction imaging was conducted at room temperature with 10 min exposure time using CrystalisPro software version 1.171.40_64.14a (Rigaku Oxford Diffraction).

### 4.4. Scanning Electron Microscopy (SEM)

GFP-hnRNPK hydrogel was mounted onto a coverslip coated with poly-_L_-lysine. The hydrogel was fixed using 2.5% glutaraldehyde at 4 °C for 2 h, then washed with ethanol for dehydration. The dried sample was mounted on an SEM aluminum stub with double-sided carbon tape and coated with gold. Imaging was carried out using a variable-pressure field-emission scanning electron microscope (1555, Carl Zeiss; Centre for Microscopy, Characterisation and Analysis, University of Western Australia) at 10 kV.

### 4.5. Fluorescence Microscopy

A 20 µL GFP-hnRNPK sample was prepared in the presence of 10% PEG 8000 (50 mM HEPES, 300 mM KCl, pH 7.4, 10% PEG 8000) in a 384-well microplate. The plate was imaged immediately using an Eclipse Ti2 inverted research microscope (Nikon) at room temperature. The images obtained from the fluorescence microscope were processed using FIJI software 1.52p [65].

### 4.6. Saturation Concentration Determination by Centrifugation

In order to evaluate the impact of PEG 8000 and salt concentrations on the phase separation of eGFP-hnRNPK, three conditions containing 10% PEG 8000 in combination with different KCl concentrations (300 mM, 150 mM, or 100 mM KCl) were tested compared to a negative control using size-exclusion buffer to dilute eGFP-hnRNPK sample. After dilution, protein samples were centrifuged at 10,000× *g* for 10 min at 4 °C to separate the condensed phase from the dilute phase. The eGFP-hnRNPK absorbance at 280 nm of the dilute phase was measured using a NanoDrop spectrophotometer (Thermo Scientific). The eGFP-hnRNPK concentration was then calculated using the extinction coefficient at 280 nm of 41,830 M^−1^cm^−1^ obtained from the ExPasyProtparam online tool [66]. Each measurement was carried out in triplicate.

### 4.7. Dynamic Light Scattering (DLS)

Protein and solvents were filtered through 0.22 µm syringe filters (Merck Millipore). Solvents were used later to rinse plastic disposable cuvettes before use, while protein was subsequently centrifuged at 16,000× *g* at 4 °C for 15 min to eliminate any insoluble aggregates or contaminants. Plastic disposable cuvettes were pre-cooled on ice to contain samples measured at 4 °C. The cuvettes were sealed by parafilm after being filled with samples to avoid introduction of dust or contaminants into the solution. To measure the change in size caused by thermal alteration, the same cuvettes were equilibrated at 25 °C for at least 30 min before remeasurement. Each measurement was performed using at least three replicates. Dynamic light scattering (DLS) data were recorded using a Malvern ZetasizerNanoZS DLS (Malvern Instruments, Malvern, UK) and Zetasizer software v7.11. Scattering light was recorded at a 173° backscatter optic to minimize the effect of multiple scattering, dust, and supramolecular contamination on the measurement.

### 4.8. Size-Exclusion Chromatography Coupled with Light Scattering (SEC-LS)

SEC-LS measurements of hnRNPK were performed at room temperature. The SEC-LS system includes a Superdex 200 Increase 10/300 column (GE Healthcare, Chicago, IL, USA) for sample separation, ViscotekGPCmax liquid chromatography system, UV detector 2600 (recording at 280 nm), and a TDA 305 (Malvern Panalytical) (measuring RALS, and RI signals). The sample vial holder temperature was set to 4 °C.

### 4.9. Small-Angle X-Ray Scattering (SAXS)

SAXS data were collected at the SAXS/WAXS beamline of the Australian Synchrotron coupled with size-exclusion chromatography (SEC- SAXS) [67]. The setup includes an inline Superdex 200 Increase 5/150 column (GE Healthcare) attached to a Shimadzu HPLC system. Samples of hnRNPK were maintained at 4 °C or 25 °C during measurement. Samples were injected into the SEC-SY-SAXS system and eluted directly into a continuous-flow cell capillary, where scattering data were collected using a Pilatus 1M detector [68]. Data were reduced using ScatterBrain [69]. Primary data processing was performed using the ATSAS 3.0.3 software package [70]. SAXS data analysis statistics are presented in Table 1.

### 4.10. Small-Angle Neutron Scattering (SANS)

SANS measurements were carried out at the Australian Nuclear Science and Technology Organization (ANSTO) OPAL reactor, using the QUOKKA neutron-scattering instrument. hnRNPK samples were loaded into quartz Hellma QS-120 cells with a 2 mm path length for measurement. Data were collected using a neutron wavelength of 6 Å and a wavelength resolution of ∆*λ*/*λ* = 0.1. Data were reduced using Igor Pro software 7.08 (Wavemetrics, Lake Oswego, OR, USA). Scattering data were processed using SASView 4.2.2 software [71]. Further analysis was performed using PRIMUS [72], GNOM [73], and DAMMIF [36] from the ATSAS 3.0.3 package [70].

### 4.11. Transmission Electron Microscopy (TEM)

For grid preparation, 5 µL of the diluted sample was adsorbed to glow-discharged 400-mesh carbon-coated Parlodion copper grids washed with three drops of water, incubated with 3 μL of tobacco mosaic virus-containing solution (kindly provided by R. Diaz Avalos, Institute of Molecular Biophysics, Florida State University), further washed with two drops of water, and finally stained with two drops of 2% (*w*/*v*) uranyl acetate. Grids were imaged using a Tecnai12 transmission electron microscope (FEI, Eindhoven, the Netherlands) operating at 120 kV. Electron micrographs were recorded on a 4096-by-4096 pixel charge-coupled device camera (TVIPS F416) at a nominal magnification of ×67,000, yielding a final pixel size of 0.198 nm on the specimen level. Micrographs were processed by reference-free alignment on manually selected particles from micrographs using the EMAN2 2.9 image processing package [74]. The extracted particles were aligned and classified by multivariate statistical analysis, yielding two-dimensional (2D) class averages.

## Figures and Tables

**Table 1:** Parameters of SEC-LS for hnRNPK at room temperature and 4 °C.

	Peak 1				Peak 2			
	Elution Volume (mL)	Measured MM (kDa)	MM Distribution (kDa)	Stoichiometry	Elution Volume (mL)	Measured MM (kDa)	MM Distribution (kDa)	Stoichiometry
hnRNPK at 4 °C	12.4	101.3	49.0–152.9	1.98:1	14.0	59.1	54.0–73.0	1.16:1
hnRNPK at RT	12.5	146.9	114.8–282.4	2.88:1	13.6	78.0	51.0–138.0	1.53:1

**Table 2:** SANS- and SEC-SAXS-derived structural parameters of hnRNPK.

Sample	hnRNPK at 25 °C	hnRNPK at 4 °C	Hydrogenated hnRNPK Monomer	Hydrogenated hnRNPK Aggregate
**Data Collection Parameters**				
Instrument	Australian Synchrotron SAXS/WAXS beamline		QUOKKA, SANS beamline, ANSTO	
*q* range (Å^−1^)	0.0031–0.0751	0.0080–0.4800	0.0060–0.42	0.0060–0.0824
Concentration (mg/mL)	0.82	6.20	1.1 (unmatched); 26.6 (total)	28.9 (unmatched)
Sample temperature (°C)	22 °C	4 °C	20 °C	20 °C
**Structural Parameters**				
*I(0)*, cm^−1^, from Guinier	0.1036 ± 0.001000	0.0062 ± 0.000075	0.0692 ± 0.001500	34.1000 ± 0.000880
*R_g_*, Å, from Guinier	106.0 ± 1.40	39.4 ± 2.96	28.8 ± 0.90	88.48 ± 0.01
*I(0),* cm^−1^, from *P(r)*	0.109300 ± 0.0010280	0.006347 ± 0.0002068	0.074000 ± 0.0011000	34.120000 ± 0.0008103
*R_g_,* Å, from *P(r)*	113.9 ± 1.710000	44.0 ± 2.695000	32.9 ± 0.600000	88.71 ±0.004509
*D*_max_, nm	42.5	18.7	11.2	28.6
*P(r)* Quality estimate	0.6733	0.6411	0.5840	0.6500
**Molecular Mass Estimation by Bayesian Probability**				
Estimated Molecular mass, M_r_ (kDa)	873.1	54.4	67.5	1267.9
MM Probability, %	32.6	21.5		
Credibility Interval (kDa)	614.5, 1013.1	46.2, 63.1		
Credibility Interval Probability, %	93.2	93.1		
Calculated M_r_from sequence (kDa) †	51.0	51.0		
Estimated Ratio/state ^†^	Aggregate	Monomer	Monomer	Aggregate
**DAMMIF (default parameters, 20 calculations)**				
Symmetry, anisotropy assumptions	P1, none	P1, none	P1, none	P1, none
χ^2^	0.2892	0.2594	0.6888	-
NSD (standard deviation)	1.094 (0.078)	0.651 (0.037)	0.893 (0.048)	-
Resolution (from SASRES) (Å)	97 ± 7	39 ± 3	40 ± 3	-

## Data Availability

SAXS and SANS data are in progress of submission to SASBDB.

## Supplementary Materials

The following supporting information can be downloaded at: https://www.mdpi.com/article/10.3390/ijms26031356/s1.

## Abbreviations

The following abbreviations are used in this manuscript:

hnRNPK	heterogeneous nuclear ribonucleoprotein K
DLS	dynamic light scattering
SEC-LS	size-exclusion chromatography coupled with light scattering
SAXS	small-angle X-ray scattering
SANS	small-angle neutron scattering
TEM	transmission electron microscopy
SEM	scanning electron microscopy
LCD	low-complexity domain
KH	K-homologous
KI	K-interactive
ALS	amyotrophic lateral sclerosis
DTT	dithiothreitol
MM	molecular mass
*C*_sat_	saturation concentration
