# Supplementary material for: Structural Characteristics and Properties of the RNA-Binding Protein hnRNPK at Multiple Physical States
Source: Int J Mol Sci. 2025 Feb 5;26(3):1356. doi: 10.3390/ijms26031356 (PMC11818384; doi:10.3390/ijms26031356)
Supplement: Supplementary file 1 [file ijms-26-01356-s001.zip › ijms-3418668-supplementary.pdf]

Supplemental Figure S1

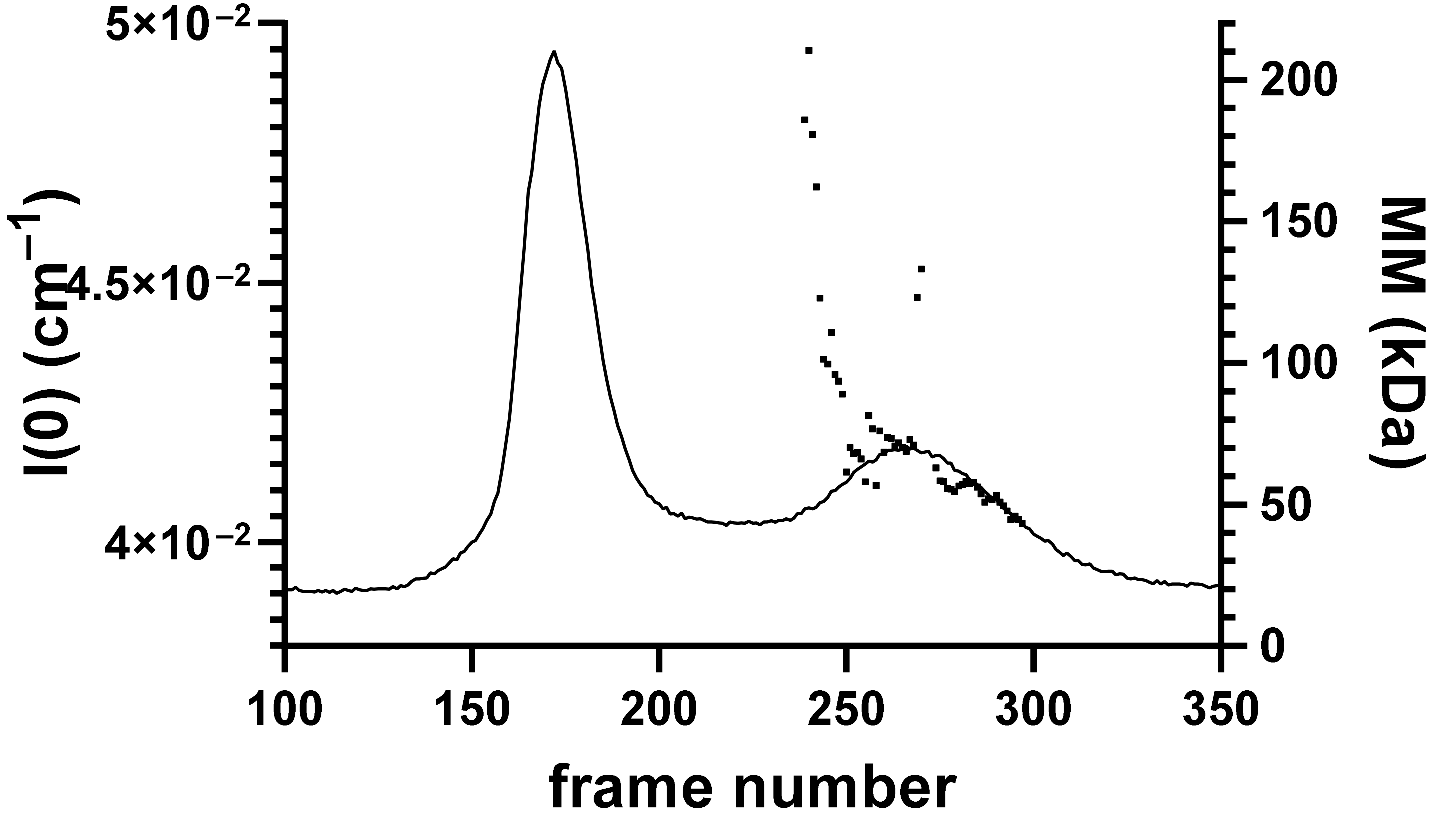

Supplemental Figure Legends

Figure S1 Scattering profile of hnRNPK at 4 °C. Plot of $I(0)$ trace and molecular mass distribution from SEC-SAXS of hnRNPK at 4 °C. The scattering data were selected from frames 360–371 for buffer and 239–297 for hnRNPK sample.
